# Supplementary material for: Fishery catch records support machine learning-based prediction of illegal fishing off US West Coast
Source: PeerJ. 2023 Oct 19;11:e16215. doi: 10.7717/peerj.16215 (PMC10590572; doi:10.7717/peerj.16215)
Supplement: Supplemental Information 1 [file peerj-11-16215-s001.docx]

The NOAA National Marine Fisheries Service has required VMS on the open access groundfish fleet operating in the EEZ (3-200 nm offshore, outside state waters) since 2008 to monitor compliance around conservation areas (50 CFR §660.14). The Pacific Fishery Management Council and the jurisdiction provided through the Magnuson Steven Act, ensures the ability of NMFS to enforce fishing behaviors and in 2020, the transmission interval for VMS for West Coast fleets was increased from one position per hour to a position every 15 minutes, 24 hours a day; some exemptions / exceptions exist (50 CFR §660.14). Vessels with federal limited entry permits are required to have VMS, regardless of whether they fish within federal water or exclusively within state water. However, for those vessels participating in the open access fishery, fishing location became a factor in whether VMS was required.

VMS requirements mandate that fishers purchase the VMS units and pay all data transmission fees, though some reimbursements are available for the purchase of VMS units. Thus, with VMS requirements, it became a business decision for open access vessels (those without Federal Limited Entry Permits) whether it made financial sense to pay the costs of VMS, in order to have access to groundfish in the EEZ; or if they wanted to avoid the VMS requirement and fish exclusively in state waters.

Another vessel tracking option that NOAA would use for enforcement if the data were more comprehensive is AIS (automatic identification systems), which is mandated by the Coast Guard instead of by NOAA. AIS is required on commercial fishing vessels 65' or greater. This is a relatively small percentage of the open access groundfish fleet. Smaller vessels often choose not to use AIS because of the expense of the units or for privacy concerns. Because AIS data are visible to other fishers as well as to NOAA enforcement and the Coast Guard, many vessel prefer not to share their locations.
