# Supplementary figures and images for: Fishery catch records support machine learning-based prediction of illegal fishing off US West Coast
Source: PeerJ. 2023 Oct 19;11:e16215. doi: 10.7717/peerj.16215 (PMC10590572; doi:10.7717/peerj.16215)

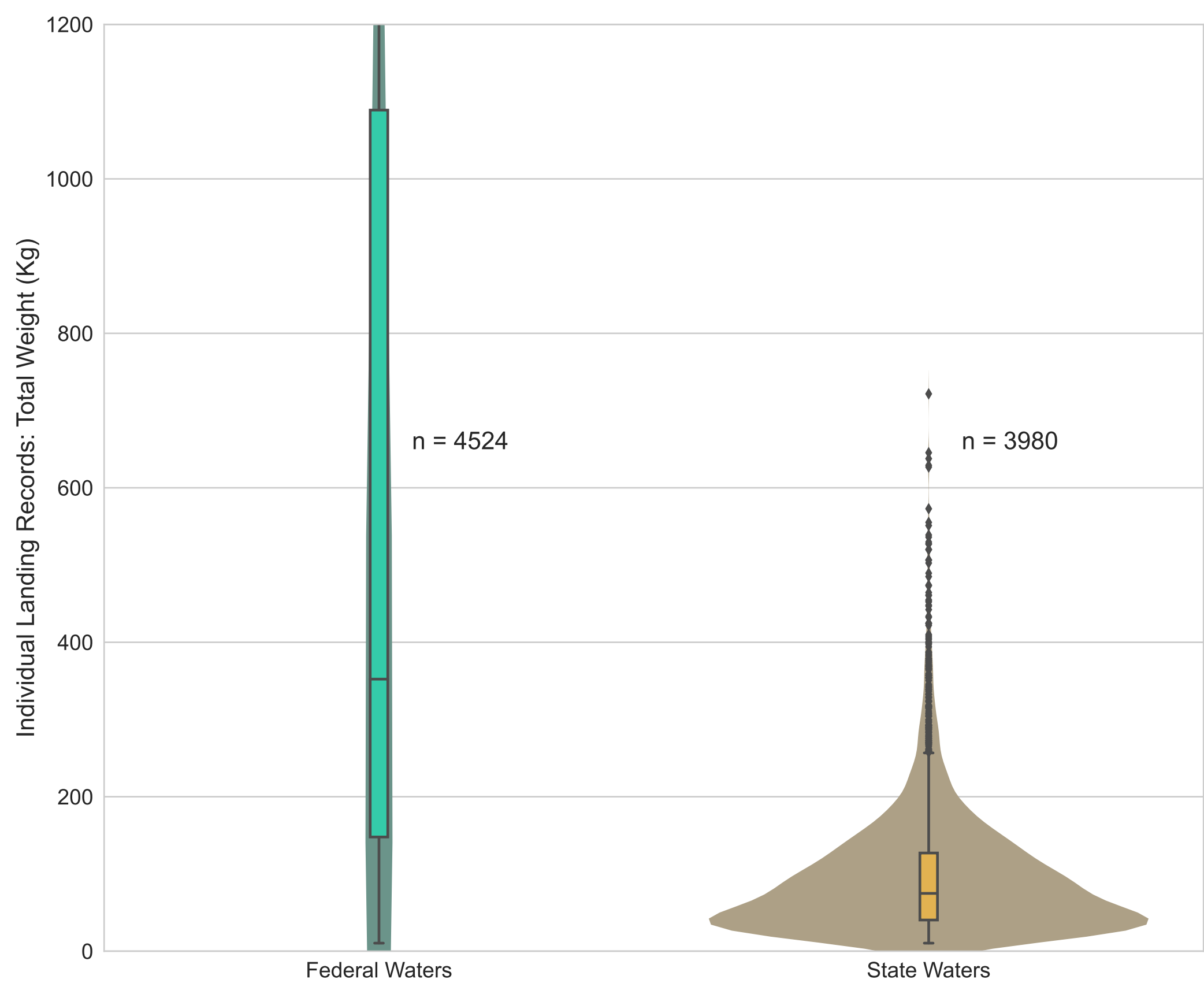

Supplement: Supplemental Information 2 — The inner boxplots show the upper and lower quartiles of the landed weights with the whiskers extending to show the rest of the distribution along with outliers as points. [file peerj-11-16215-s002.pdf]

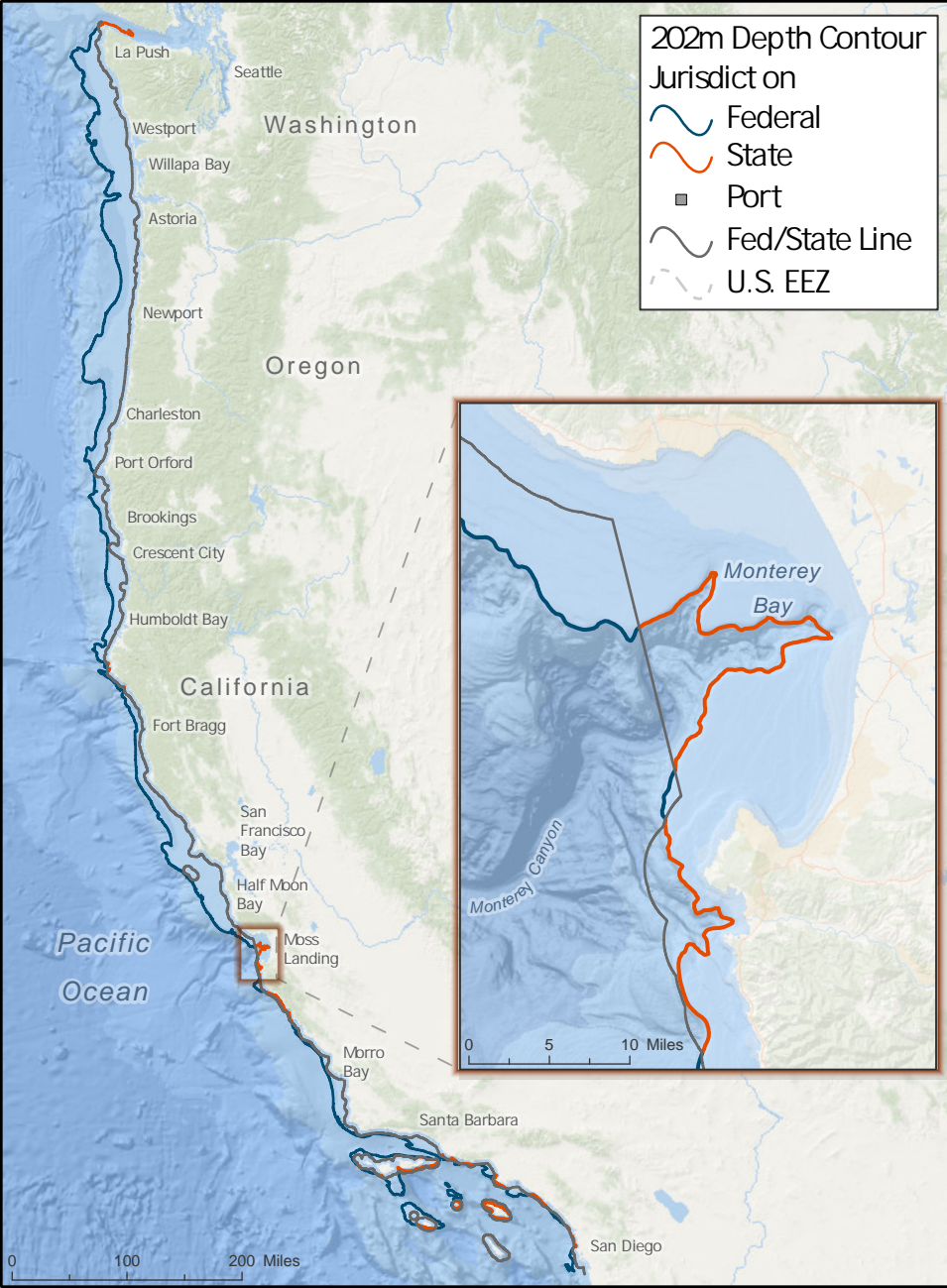

Supplement: Supplemental Information 3 — Colors indicate Federal (blue) and State (red) jurisdiction of the depth contour. [file peerj-11-16215-s003.pdf]

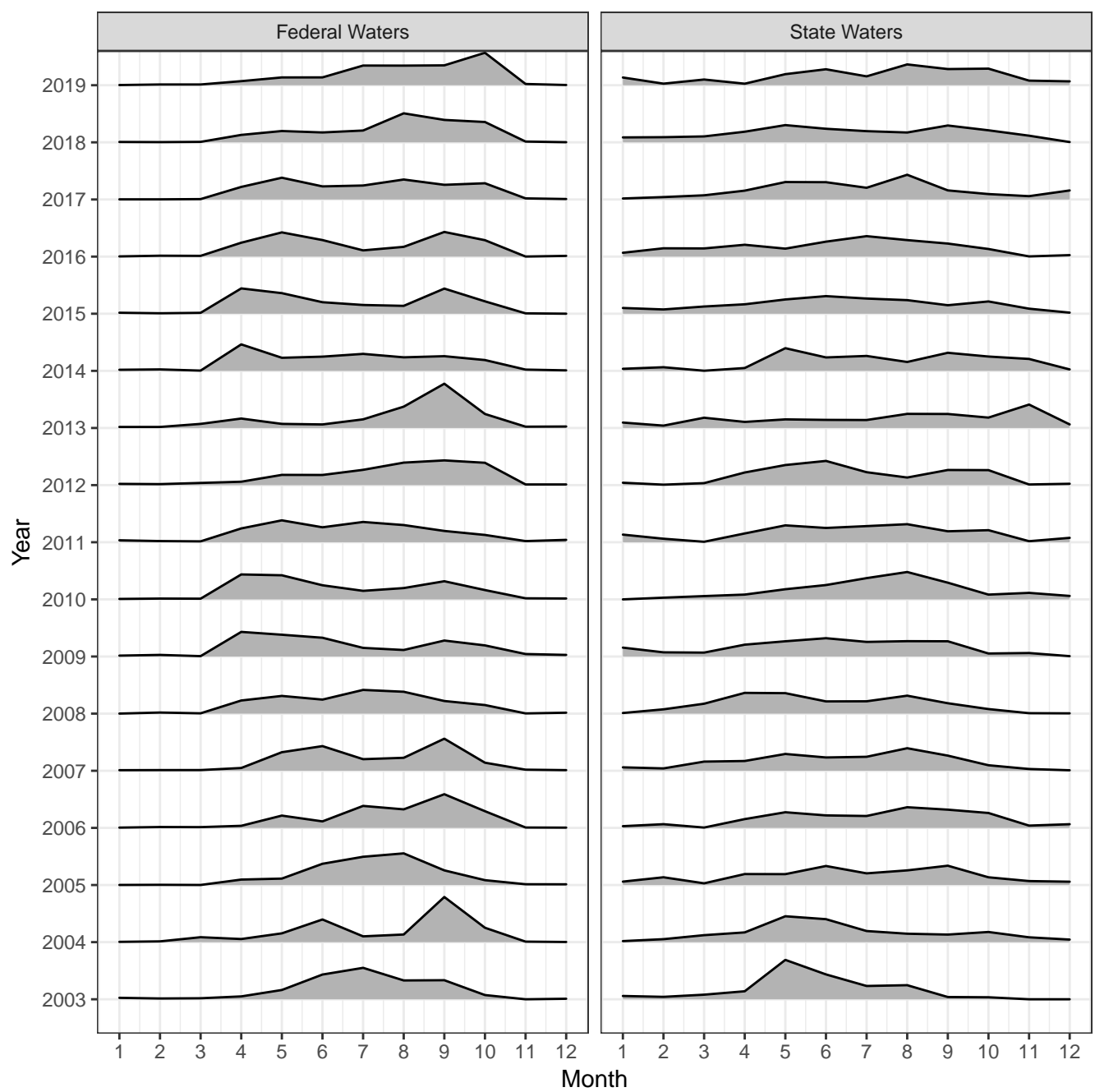

Supplement: Supplemental Information 4 — While mode shifts occur across years, the general distributions across years for federal (left) and state (right) waters are relatively consistent and variability that is observed may be driven somewhat by selection for observer coverage. [file peerj-11-16215-s004.pdf]

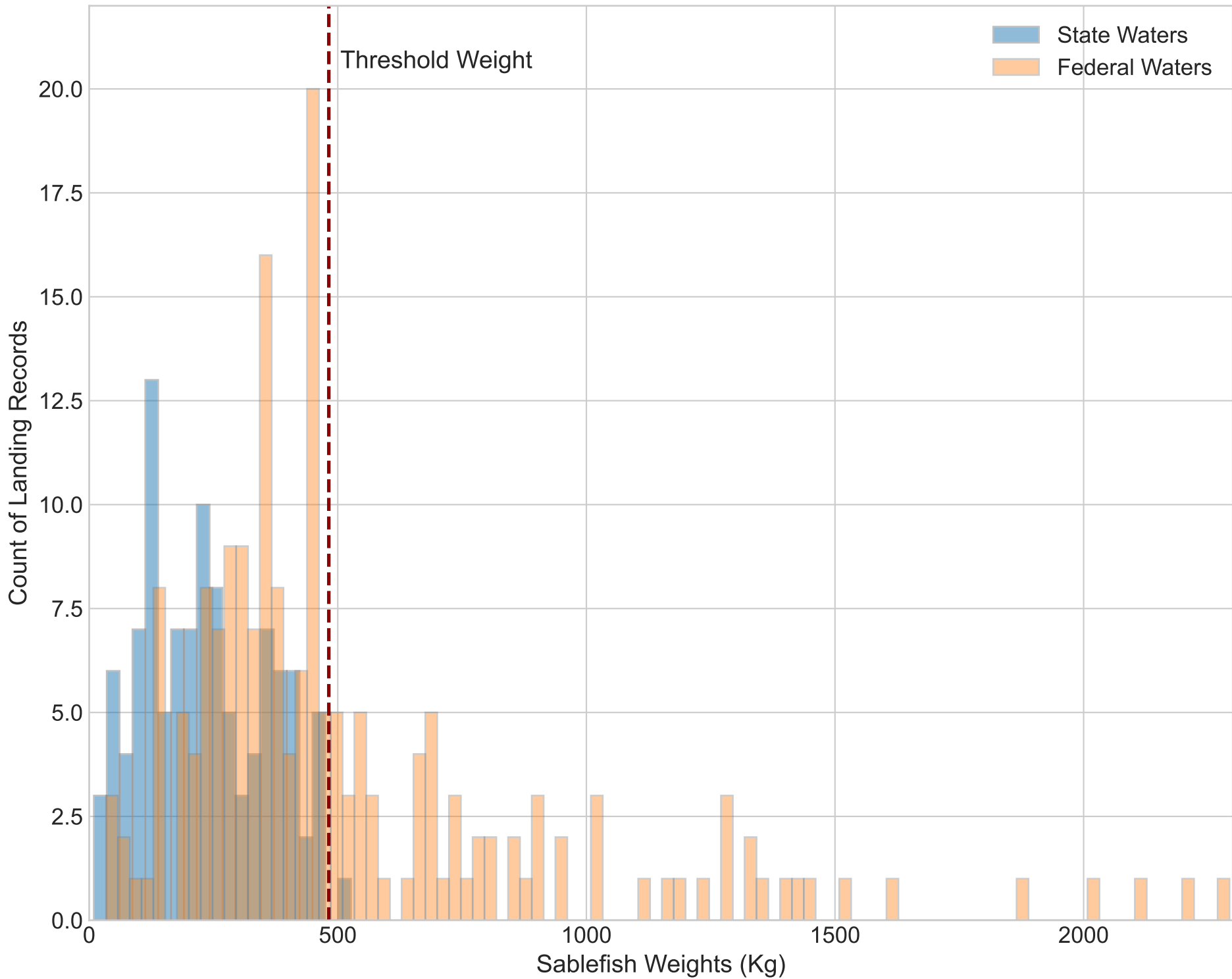

Supplement: Supplemental Information 5 [file peerj-11-16215-s005.pdf]

ROC curve:  
Random Forest and Gradient Boosting Models

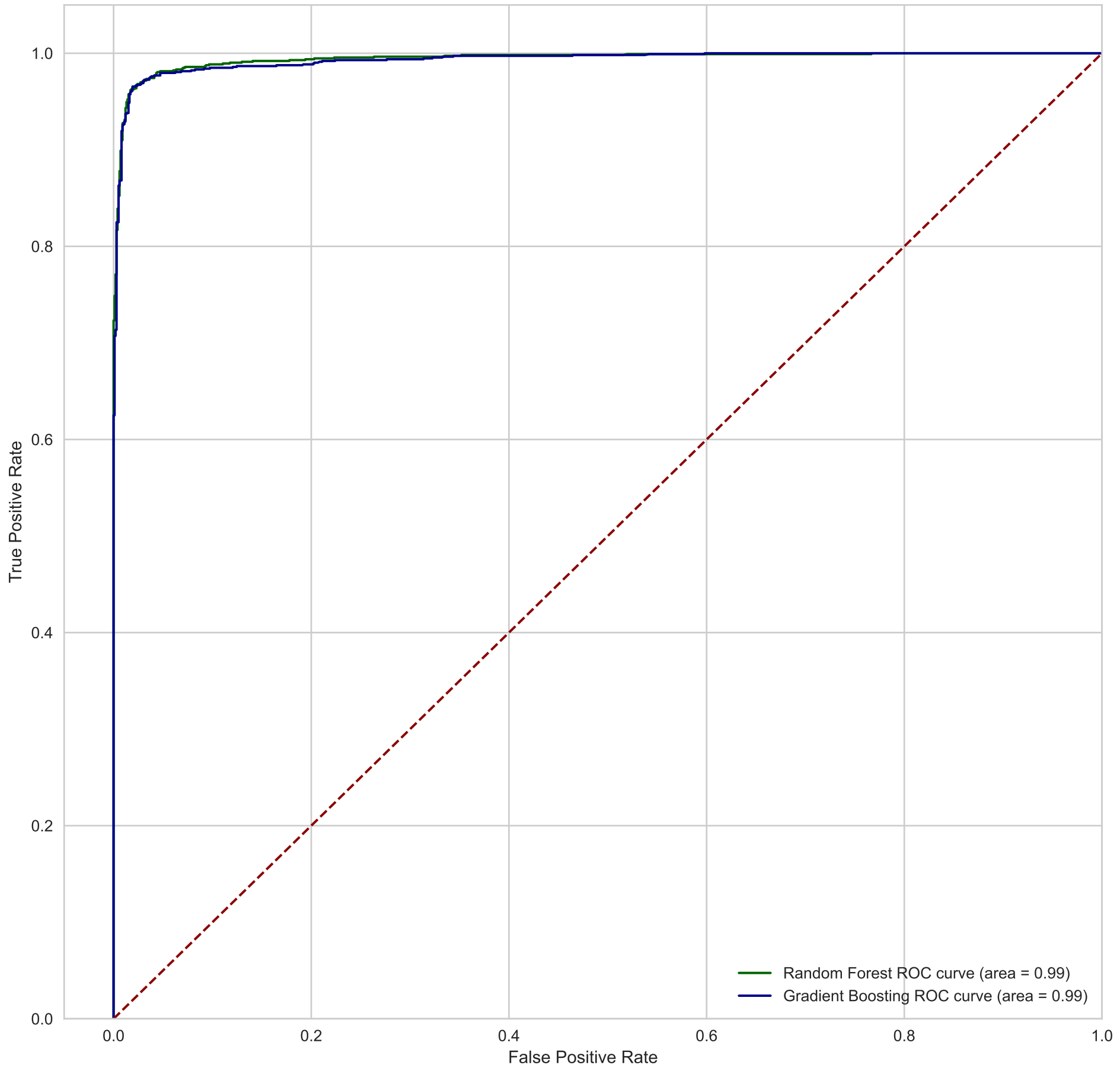

Supplement: Supplemental Information 6 [file peerj-11-16215-s006.pdf]
