# Supplementary material for: Fishery catch records support machine learning-based prediction of illegal fishing off US West Coast
Source: PeerJ. 2023 Oct 19;11:e16215. doi: 10.7717/peerj.16215 (PMC10590572; doi:10.7717/peerj.16215)
Supplement: Supplemental Information 7 — Confidential columns and data have been omitted. [file peerj-11-16215-s007.pdf]

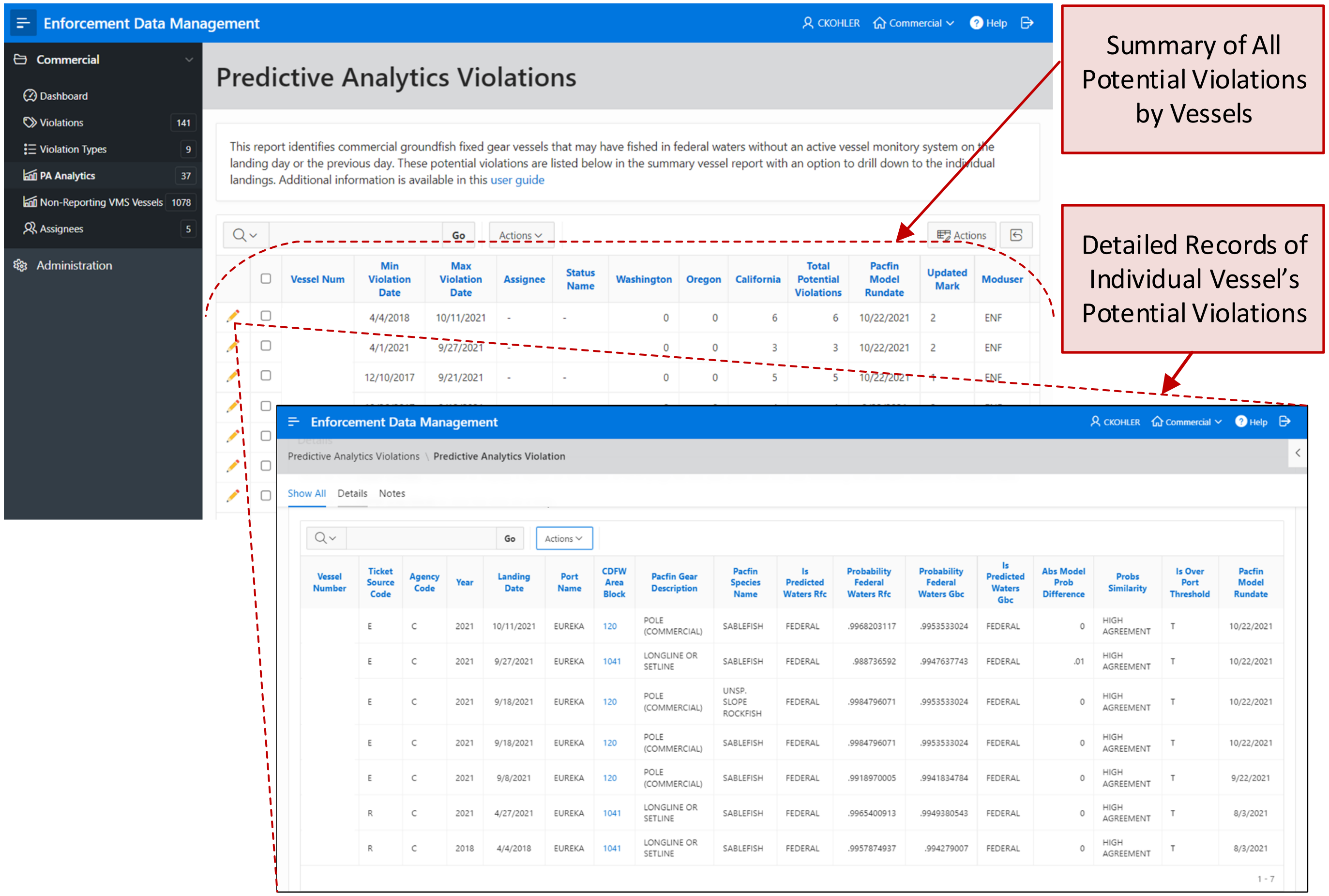
Enforcement Data Management
CKOHLER
Commercial
Help
Commercial
Dashboard
Violations 141
Violation Types 9
PA Analytics 37
Non-Reporting VMS Vessels 1078
Assignees 5
Administration
Predictive Analytics Violations
This report identifies commercial groundfish fixed gear vessels that may have fished in federal waters without an active vessel monitory system on the landing day or the previous day. These potential violations are listed below in the summary vessel report with an option to drill down to the individual landings. Additional information is available in this user guide
Summary of All Potential Violations by Vessels
Detailed Records of Individual Vessel's Potential Violations
Predictive Analytics Violations \ Predictive Analytics Violation
Show All
Details
Notes
